# Pest scenario of *Helicoverpa armigera* (Hub.) on pigeonpea during future climate change periods under RCP based projections in India

**DOI:** 10.1038/s41598-023-32188-1

**Published:** 2023-04-26

**Authors:** M. Srinivasa Rao, C. A. Rama Rao, B. M. K. Raju, A. V. M. Subba Rao, D. L. A. Gayatri, Adlul Islam, T. V. Prasad, M. Navya, K. Srinivas, G. Pratibha, I. Srinivas, M. Prabhakar, S. K. Yadav, S. Bhaskar, V. K. Singh, S. K. Chaudhari

**Affiliations:** 1 ICAR—Central Research Institute for Dryland Agriculture (CRIDA), Hyderabad, 500 059 India; 2 ICAR—Natural Resources Management (NRM), Krishi Anusandhan Bhavan, Pusa, New Delhi India; 3 Principal Scientist (Entomology), ICAR—Central Research Institute for Dryland Agriculture (CRIDA), Hyderabad, Telangana 500059 India

**Keywords:** Plant sciences, Climate sciences

## Abstract

Gram pod borer, *Helicoverpa armigera* (Hub.) is the major insect pest of pigeonpea and prediction of number of generations (no. of gen.) and generation time (gen. time) using growing degree days (GDD) approach during three future climate change periods viz., Near (NP), Distant (DP) and Far Distant (FDP) periods at eleven major pigeonpea growing locations of India was attempted. Multi-model ensemble of Maximum (Tmax) and Minimum (Tmin) temperature data of four Representative Concentration Pathways viz., RCP 2.6, 4.5, 6.0 and 8.5 of Coupled Model Inter comparison Project 5 (CMIP5) models was adopted here. The increase in projected Tmax and Tmin are significant during 3 climate change periods (CCPs) viz., the NP, DP and FDP over base line (BL) period under four RCP scenarios at all locations and would be higher (4.7–5.1 °C) in RCP 8.5 and in FDP. More number of annual (10–17) and seasonal (5–8) gens. are expected to occur with greater percent increase in FDP (8 to 38%) over base line followed by DP (7 to 22%) and NP (5to 10%) periods with shortened annual gen. time (4 to 27%) across 4 RCPs. The reduction of crop duration was substantial in short, medium and long duration pigeonpeas at all locations across 4 RCPs and 3 CCPs. The seasonal no.of gen. is expected to increase (5 to 35%) with shortened gen. time (4 to 26%) even with reduced crop duration across DP and FDP climate periods of 6.0 and 8.5 RCPs in LD pigeonpea. More no. of gen. of *H. armigera* with reduced gen. time are expected to occur at Ludhiana, Coimbatore, Mohanpur, Warangal and Akola locations over BL period in 4 RCPs when normal duration of pigeonpeas is considered. Geographical location (66 to 72%), climate period (11 to 19%), RCPs (5–7%) and their interaction (0.04–1%) is vital and together explained more than 90% of the total variation in future pest scenario. The findings indicate that the incidence of *H. armigera* would be higher on pigeonpea during ensuing CCPs in India under global warming context.

## Introduction

Climate change is a major threat to sustainable agriculture^1^ and its impact on incidence of insect pests is an important dimension of overall impact on agriculture. Understanding of the complex, spatially variable and species-specific effects of climate change is essential to develop an appropriate pest management strategy. Complete comprehension and quantification of insect herbivore-climate relationship is the crux of the pest management strategy as insect growth and development is driven by the climate. IPCC^2^ summary report projected that global mean surface temperatures would rise in the range of 1–3 °C by the end of this century. When averaged over 2081–2100, the global surface temperature is very likely to be higher by 1.0 to 1.8 °C in the very low, 2.1 to 3.5 °C in the intermediate and 3.3 to 5.7 °C in the very high GHG emission scenarios in comparison with the average between 1850 and 1900. The projected temperature rise for India is in the range of 1.7–2.0 °C by 2030s & 3.3–4.8 °C by 2080s over preindustrial period^3^. Further, the average temperature over India is projected to rise by approximately 4.4 °C relative to the recent past (1951–2014) under RCP 8.5 scenario^4^.

The variation of temperature is prominent and non-uniform among different areas, provinces, locations and negative impacts have been projected on various crops^5^. Bale et al. (2002)^6^ reported that insect development and its distribution is mostly temperature driven. Under warming conditions, early infestation, increased survival of insects and more crop damage would occur. About two to four-fold increase in herbivory^7^ and severity in pest outbreak^8^ are reported as a result of climate change. Thermal requirement of insects is species-specific, and the role of temperature is vital and prominent on development of each insect species^9^. Degree days are the amount of heat required for an organism to develop within certain life stages and growing degree day (GDD) is the summation of heat units that accumulated above base temperature during a 24-h period. Degree days concept is an effective way to explain shifts in phenology, arising from climate change over space and time^10^ and it is a very strong, integrative measure of the spatio-temporal variation of temperature that imposes the thermal limits within which insect species grow^11^.

Temperature effects on insect pests are species-specific and elaborative studies were conducted earlier. The effect of temperature on the insects is direct and dependent on the amount of time they are exposed, as that causes physiological changes that aid survival^12^. The development and distribution of insects are influenced by their thermal thresholds; though thermal requirements vary with species; climate warming can expand the survival limits^13^ and often may lead to higher number of generations (no. of gen.) in a year or a season. Increased adult moth activity^14^, increased number of generations^15^, higher incidence^16^, higher population growth rate^17^ were the effects of increased temperature on insect biology.

Temperature is crucial in deciding the insect population growth rate and development and further reflected in number of generations which indicates the number of reproductive events in a given period (calendar year/growing season). It affects the crop-insect interactions, implying the level of incidence indirectly. More generations not only promote the population growth but also hastens the evolutionary process and adaptation to climate^18^. Increased temperature leads to higher developmental rate and in turn increases the voltinism of the pest. The possibility of number of generations in a year is a function of temperature, precipitation and availability of host^19^. Insects are sensitive to increased temperature^20^ and its impact on insect leads to advancement of phenology/ life stages. In case of insect pests, longer developmental duration at low temperatures and a shorter developmental duration at high temperatures was documented earlier^21^.

Pigeonpea (*Cajanus cajan* L.), a legume crop belonging to the tribe *Phaseoleae,* is one of the most important grain legumes. It has 21% of dietary protein^22^ in seeds and is rich in minerals like phosphorus, magnesium, iron, calcium, sulphur and potassium^23^.The global area under cultivation of pigeonpea is 5.7 Mha with a productivity of 861.3 kg/ha and overall production of 4.91 Mt per annum over the triennium of 2017–2020^24^. India is the major producer (78%). Myamnar, Malawi, Tanzania, Kenya and Uganda also have significant area under pigeonpea. Among the pigeonpea growing countries, India ranked 1 in area and production representing around 82% of world pigeonpea area and 78% of total production. Pigeonpea is grown in all states of India for various purposes. In the triennium of 2017–2020, pigeonpea was cultivated on an average area of 4.6 Mha with an average production of 3.8 Mt per year and with a productivity of 824 kg per hectare^25^ in India.

Among the several insect pests (about 150) that attack pigeonpea, gram pod borer *Helicoverpa armigera* (Hub.) is a major insect pest which causes up to 20–30% yield losses^26^. Prediction of ensuing pest scenarios using temperature data based on climate projections of Coupled Model Inter comparison Project 3 (CMIP3)^27^ was attempted by several research workers^28–30^. These climate projections are generally with more accuracy at the global scale than at smaller regional scale^31^. Recently the Coupled Model Inter-comparison Project phase 5 (CMIP5) models are available with high resolution and are more comprehensive in nature over CMIP3^32^ and these projections for India are far more reliable^3^. Individual GCM projections have uncertainty and to alleviate the uncertainty, multiple and/or ensemble of GCMs is generally preferred^33^.

Earlier, many authors^33,34^ have used the World Climate Research Program’s (WRCP’s) CMIP5 multi-model dataset for generating multi-model ensemble climate change scenarios. The advantage of the CMIP5 multi-model dataset is the accuracy of newly developed representative concentration pathways (RCPs)^3^. We adopted ensembled mean climate data here which is closer to the observed climate than any individual model. IPCC Fifth Assessment Report’s RCP based climate change projections for 4 RCPs, viz., 2.6, 4.5, P 6.0 and 8.5 are adopted for our analysis.

The present study was attempted to understand the impact of simulated scenario of increased temperatures on no. of gen. and gen. time of *H. armigera* on pigeonpea for the future CCPs using temperature data of four RCPs across eleven locations of India.

## Materials and methods

Currently, climate models are the best tools to simulate future climate change scenarios and the accuracy of temperature projections mainly depends on type of model and scenario adopted.

### Projected temperature in RCPs

We followed the IPCC AR 5 climate change projections based on emission scenarios known as RCPs (Representative Concentration Pathways). As explained earlier, ensemble multimodel climate change scenarios were obtained with bias correction and at 0.5 × 0.5° spatially disaggregated climate change projections from WRCPs CMIP5 multi-model data set. In total 32 GCM climate change projections from 23 modeling Centers/Groups were used. As different GCMs have multiple runs, we used 51, 61, 34 and 64 projections (runs) for RCP2.6, RCP4.5, RCP6.0 and RCP8.5, respectively, for simulating climate change scenarios. Srinivasa Rao et al.^34^, mentioned more about details of climate change projections and advantages of hybrid ensemble data^33,35^ in their earlier publication.

**Figure 1:**
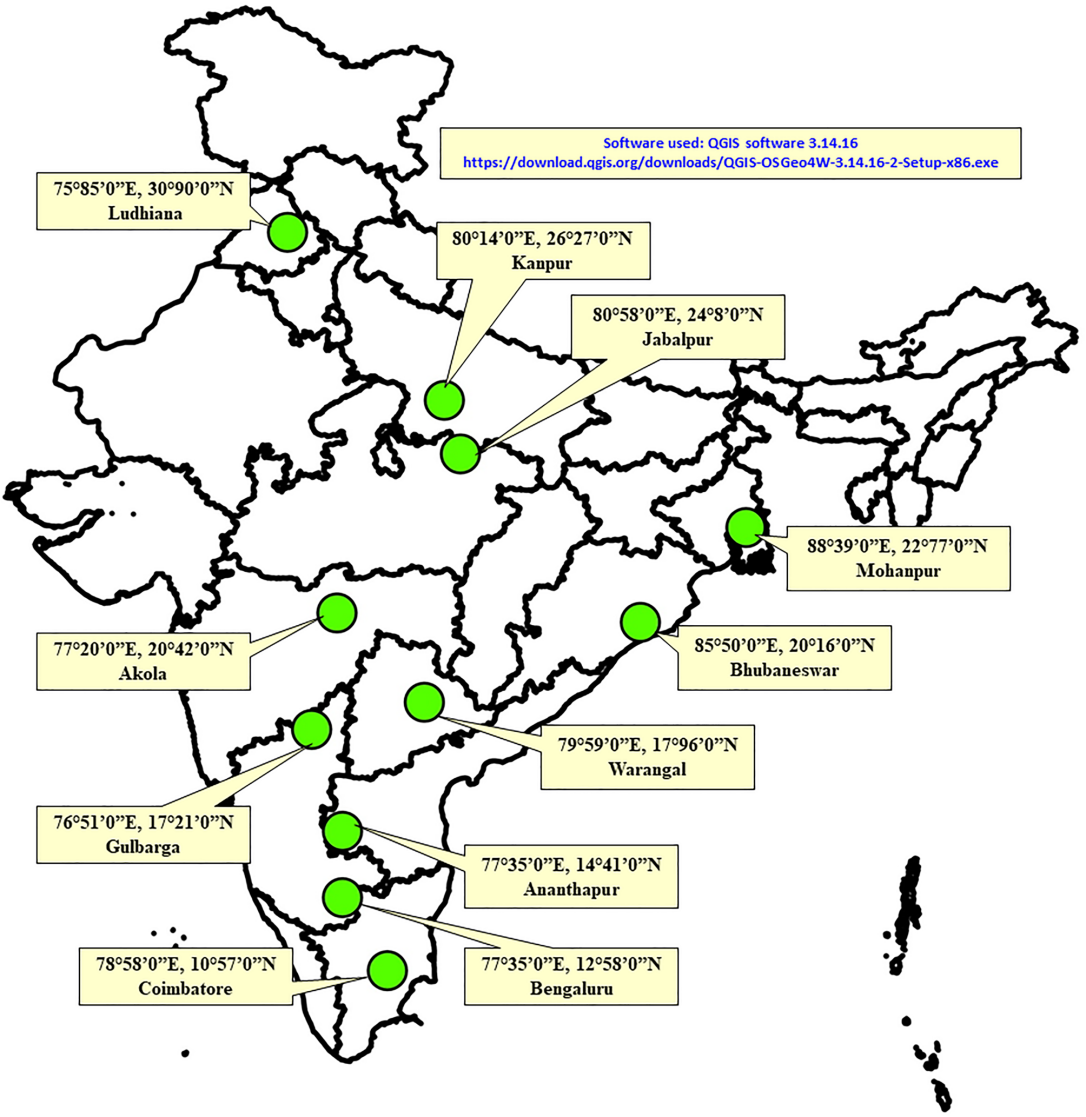
Selected pigeonpea locations of India for the study in India map using QGIS software version 3.14.16. The URL is https://download.qgis.org/downloads/QGIS-OSGeo4W-3.14.16-2-Setup-x86.exe.

In this study, climate change scenarios for 4 RCPs and 3 CCPs were adopted. Climate projections were studied over three CCPs/time slice periods (TSPs) viz. NP—Near Period (2020–2039), DP—Distant Period (2040–2069) and FDP—Far Distant Period (2070–2099). Two approaches viz., calendar year (total 365 days, 1–52 Standard Weeks) and pigeonpea crop duration (150 days from 26th to 52nd Standard Weeks) were considered for predicting the number of generations (no. of gen.) and generation time (gen. time) of *H. armigera*. Minimum (Tmin) and maximum (Tmax) temperatures for 11 pigeonpea growing locations of India for the future/ensuing periods under 4 RCPs were compared over baseline period (1976–2005). The details of these eleven pigeonpea growing locations of India are- Akola (20°42’N; 77°2’E), Ananthapur (14°41’N; 77°35’E), Bengaluru (12°58’N; 77°35’E ), Bhubaneshwar (20°16’N; 85°50’E), Coimbatore (10°57’N; 78°58’E ), Gulbarga (17°21’N; 76°51’E ), Jabalpur (24°8’N; 80°58’E ), Kanpur (26°27’N; 80°14’E), Ludhiana (30°90’N; 75°85’E), Mohanpur (22°77’N; 88°39’E) and Warangal (17°96’N; 79°59’E) depicted in a map (Fig. 1) drawn using QGIS software 3.14.16 version (open-source).

### Annual & seasonal generations of *H. armigera*

Prediction of no. of gen. of *H. armigera* was done using ‘*ingen’* software^36^ which is openly accessible (http://www.nicra-icar.in/). The *ingen* software was earlier^15,29,30,34,37^ used to obtain output of estimated thermal requirements /growing degree days, insect pest generations & gen. time. Lower threshold temperature of 10 °C was adopted to calculate the Growing Degree Days (GDD) with horizontal cutoff method. Prediction of no. of gen. and gen. time of *H. armigera* was done separately for annual/ calendar year and crop/growing season.

### Estimation of crop duration and generations in RCPs

Advancement of phenological stages of crop leads to reduction of duration for maturity with increased temperature. The degree day requirement of pigeonpea varies with sowing date^38^ location^39^ and variety^40^. We calculated the degree day requirement for short-duration pigeonpea (SDP-130 days), medium-duration pigeonpea (MDP-150 days) and long-duration pigeonpea (LDP-180 days) was in the range of 2100 to 3600 DD. Here, the accumulated heat units of 1900–2700, 2100–3000 and 2450–3550 GDD for SD, MD and LD pigeonpeas respectively were considered across 11 locations for the estimation of reduction in duration. Increased temperature influences the duration of crops^41^ by causing the advancement of phenological events and in the present study, no. of gen. and gen. time of *H. armigera* was also estimated with reduced duration of pigeonpea across SDP, MDP and LDPs.

### Statistical analysis

Change in gen. time (annual and seasonal) and no. of gen. (annual and seasonal) from baseline to near period (NP), distant period (DP) and far distant period (FDP) across 4 RCPs and 11 pigeonpea growing locations were tested for their statistical significance using two sample t test. Usually it is assumed that two populations being compared are homogenous with respect to their variances which may not always be true. In view of this, Levene test was employed to establish equality of variances or otherwise. Corrective measures were taken by employing two sample *t* test for unequal variances for comparisons with heterogenous variances. Further, two sample t test becomes valid only when the distributions of the two populations being compared are normal. To ascertain this assumption, Shapiro–Wilk test was conducted. The standard Analysis of Variance (ANOVA) was adopted to partition the variation in the study parameters viz., no. of gen. and gen. time of *H. armigera* into various sources viz., location, scenario, climate period and their interactions^15,42^ and quantified their contribution to total variability. All statistical analyses were conducted using SPSS 16.0 version.

## Results

The findings of Levene’s test indicate equal variance (with *p* value > 0.05) in the groups compared in no. of gen. (annual and seasonal) and gen. time (annual and seasonal) of *H.armigera* for 515 datasets out of 528 (11 locations × 4 RCPs × 3 CCPs with 30 observations in each period × 4 variables) studied showing the homogeneity of the data. The remaining 13 datasets were compared with unequal variances to compare the means. The Shapiro–Wilk test of significance indicated the normality of no. of gen. (annual and seasonal) and gen. time (annual and seasonal).

### Rise in Tmax and Tmin

**Figure 2:**
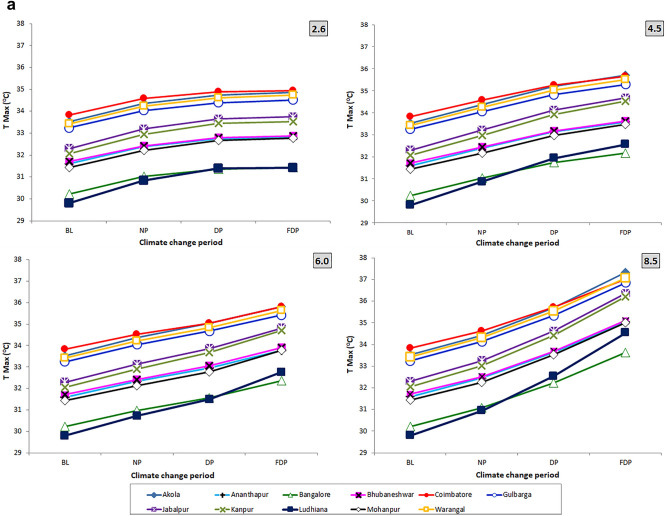

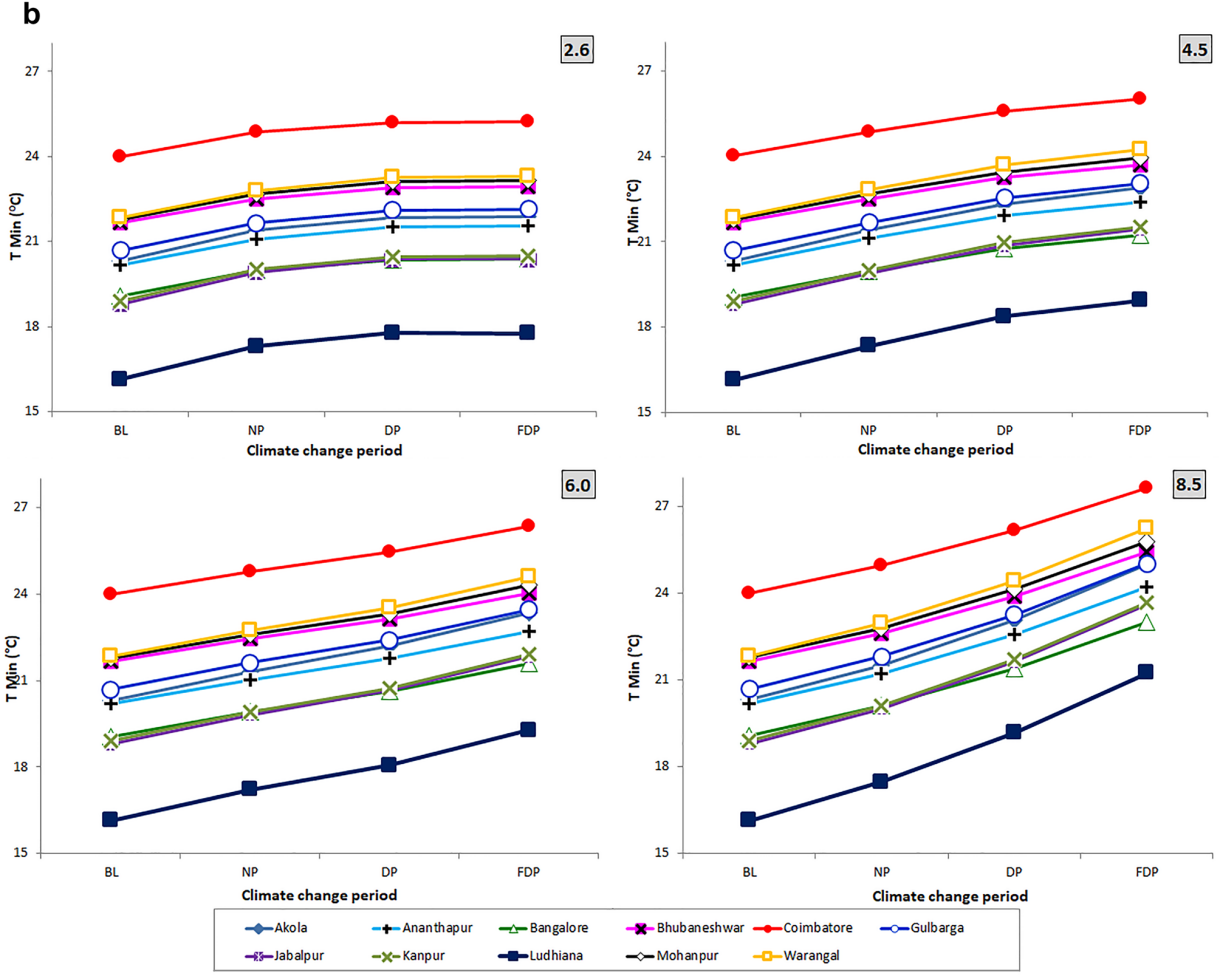
(**a**) Variation of Tmax during CCPs in 4 RCPs across different locations of India. (**b**) Variation of Tmin during CCPs in 4 RCPs across different locations of India.

The predicted Tmax and Tmin across 11 locations of India under 4 RCP scenarios when compared over baseline (Fig. 2a,b) indicated significant increase in both the temperatures in 4 RCPs. The increases were markedly higher and more evident in RCP 6.0 and RCP 8.5 during DP and FDP CCPs.

It is expected that projected temperatures (Tmax and Tmin) would follow increasing trend during 3 CCPs at 11 locations. Tmax (0.7 to 4.7 °C) and Tmin (0.8 to 5.1 °C) would increase considerably during NP, DP and FDP of 4 RCP scenarios. This trend was more evident in RCP 8.5 of FDP followed by 6.0 and 4.5 scenarios.

### Annual and seasonal generations

#### Four RCPs

**Figure 3:**
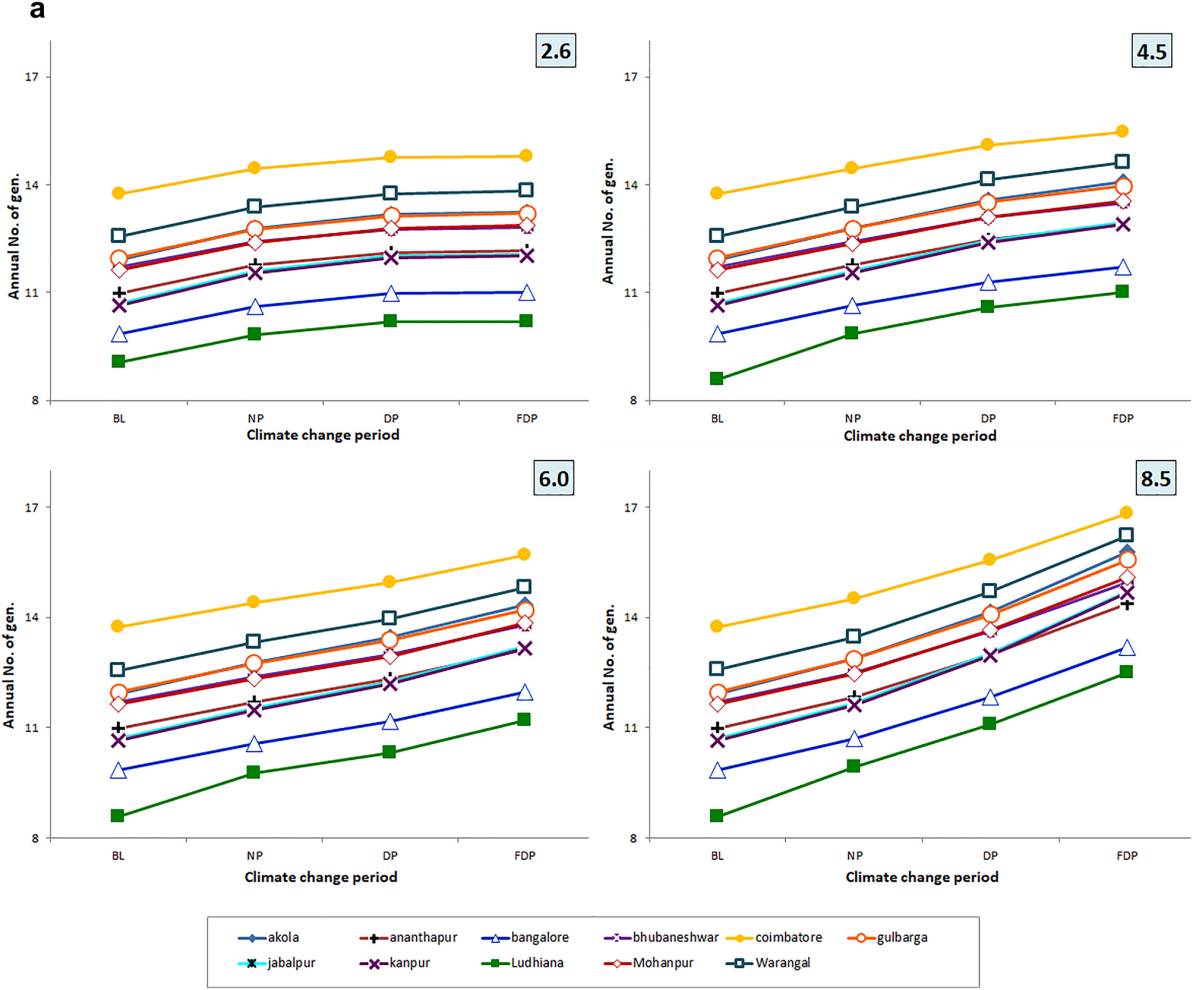

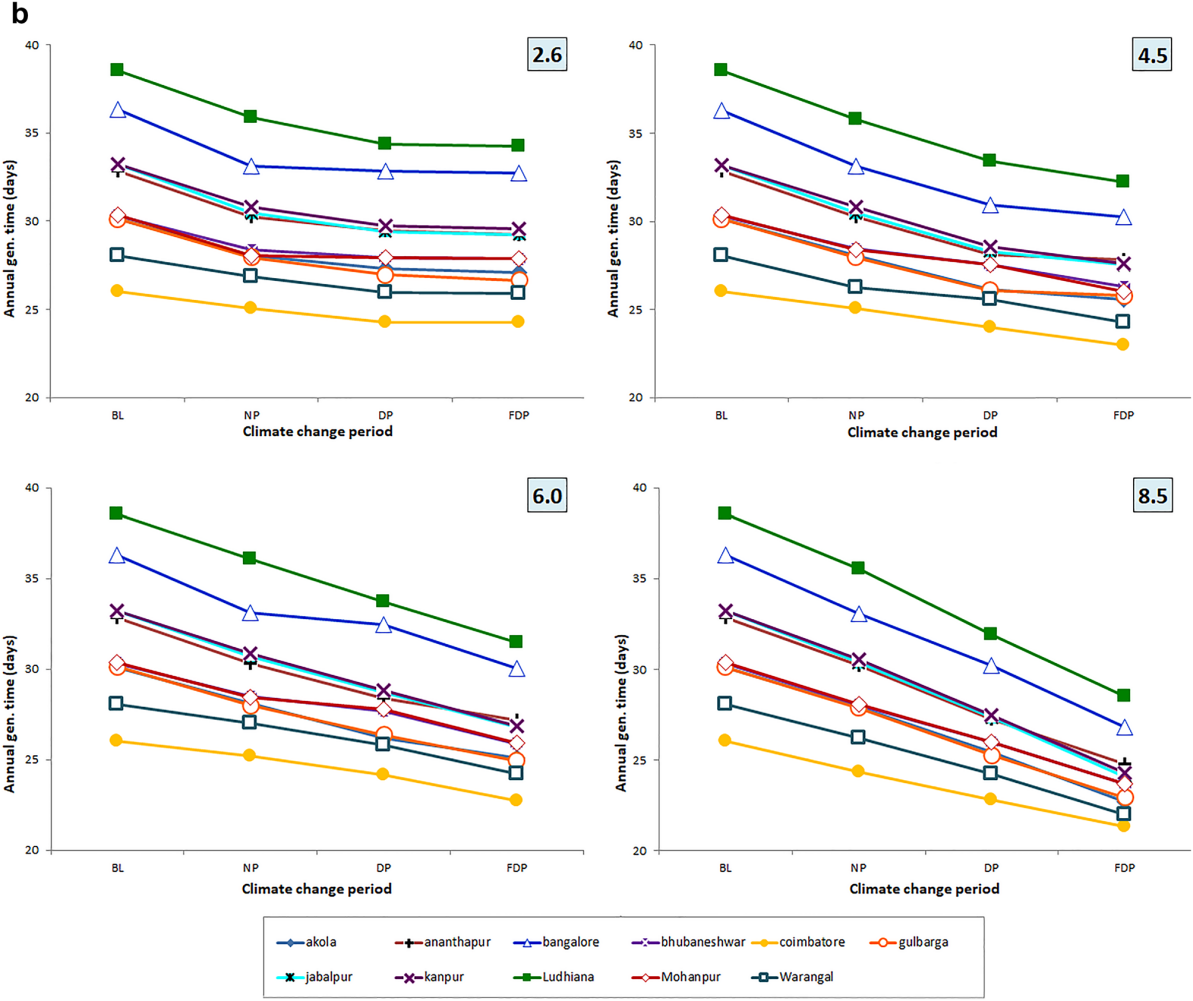
(**a**) Variation in annual no. of gen. of *H. armigera* during 3 CCPS at 11 pigeonpea growing locations of India. (**b**) Variation in annual gen. time of *H. armigera* during 3 CCPS at 11 pigeonpea growing locations of India.

Significant (F_3, 3828_ = 1768.82, *P* < 0.001) differences were observed (Table. 1) in annual num. of gen. of *H. armigera* on pigeonpea with reduced gen. time (F_3, 3828_ = 1045.33, *P* < 0.001) among RCPs with RCP 8.5 (16.83 ± 0.45; 21.31 ± 0.30) followed by RCP 6.0 (15.70 ± 0.34; 22.75 ± 0.15), RCP 4.5 (15.48 ± 0.33; 22.98 ± 0.54) over RCP 2.6 (14.79 ± 0.29; 24.25 ± 0.19). Similar trend was observed during the crop season also with significant (F_3, 3828_ = 943.68, *P* < 0.001) increase no. of gen. with shortened gen. time (F_3, 3828_ = 979.94, *P* < 0.001) in RCP 8.5 (8.49 ± 0.28; 21.21 ± 0.06) followed by RCP 6.0 (7.20 ± 0.18; 23.67 ± 0.42) RCP 4.5 (7.05 ± 0.19; 23.77 ± 0.27) and RCP 2.6 Scenarios (7.02 ± 0.18; 25.12 ± 0.73) (Fig. 3a,b).

**Table 1:** ANOVA of number of generations and generation time (Annual/Seasonal) of *H armigera* across 11 Locations, 4 RCPS and 3 CCPs.

Source	Degrees of freedom	Annual						Seasonal					
		No. of gen. (NG)			Gen. time (GT)			No. of gen. (NG)			Gen. time (GT)		
		Mean square	F	Sig	Mean square	F	Sig	Mean square	F	Sig	Mean square	F	Sig
Location	10	577.97	4802.96	0.00	2735.36	3234.60	0.00	182.24	3655.79	0.00	3305.82	4498.44	0.00
RCP	3	212.85	1768.82	0.00	883.99	1045.33	0.00	47.04	943.68	0.00	720.14	979.94	0.00
CCP	2	686.01	5700.80	0.00	2932.47	3467.68	0.00	146.22	2933.30	0.00	2491.24	3389.99	0.00
Location x RCP	30	0.14	1.17	0.24	3.03	3.59	0.00	0.06	1.29	0.13	9.77	13.30	0.00
Location x CCP	20	0.52	4.28	0.00	14.75	17.45	0.00	0.24	4.77	0.00	47.30	64.36	0.00
RCP x CCP	6	74.98	623.10	0.00	267.22	315.99	0.00	16.08	322.52	0.00	225.05	306.23	0.00
Location x RCP x CCP	60	0.06	0.50	1.00	2.35	2.78	0.00	0.02	0.48	1.00	9.50	12.93	0.00
Error	3828	0.12	–	–	0.85	–	–	0.05	–	–	0.74	–	–

### Climate change periods

**Figure 4:**
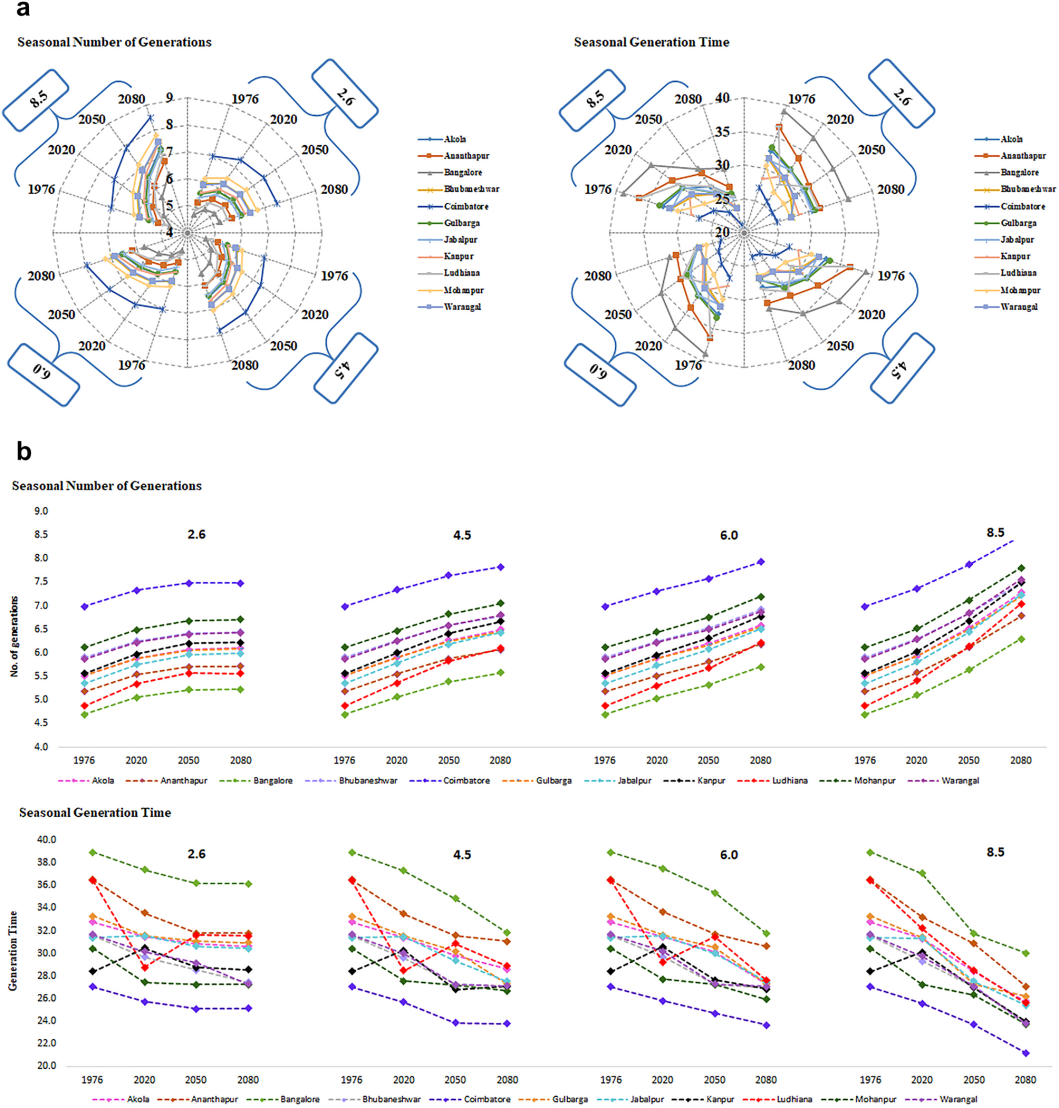
(**a**) Variation in no. of gen. and gen. time of *H. armigera* in crop season during 3 CCPs over baseline at 11 pigeonpea growing locations of India (RADAR). (**b**) Variation in no. of gen. and gen. time of *H. armigera* in crop season during 3 CCPs over baseline at 11 pigeonpea growing locations of India (PANELS).

Significant (F_2, 3828_ = 5700.80, *P* < 0.001) differences were recorded (Table. 1) in the annual no. of gen. of *H. armigera* among the CCPs with significant (F_2, 3828_ = 3467.677, *P* < 0.001) reduction in gen. time during FDP (16.83 ± 0.45, 21.31 ± 0.30) followed by DP (15.57 ± 0.35, 22.78 ± 0.10) and NP (14.52 ± 0.28, 24.33 ± 0.02) over BL period. During the crop season, (F_2, 3828_ = 2933.30, *P* < 0.001) increase in the no. of gen. (8.49 ± 0.28) with significant (F_2, 3828_ = 3389.99, *P* < 0.001) reduction in gen. time (21.21 ± 0.06) was noticed in FDP. The comparison of pest incidence between DP and NP CCPs revealed that pest incidence would be higher for DP (7.88 ± 0.21, 23.72 ± 0.37) than NP (7.37 ± 0.16, 25.56 ± 0.72) period over BL period (Fig. 4a or b or both).

### Locations

Among eleven pigeonpea growing locations, Coimbatore (16.83 ± 0.45, 21.31 ± 0.30) and Warangal (16.22 ± 0.29, 25.25 ± 0.61) would get significantly (F_10, 3828_ = 4802.96, *P* < 0.001) (Table 1) more annual no. of gen. of *H. armigera* with reduction in gen. time (F_10, 3828_ = 3234.60, *P* < 0.001) in RCP 8.5. Moderate levels were observed in Bhubaneshwar (14.96 ± 0.44, 23.67 ± 0.79) and Jabalpur (14.71 ± 0.52, 24.09 ± 0.61) locations. Bengaluru (13.19 ± 0.45, 26.83 ± 1.09) and Ludhiana (12.50 ± 0.48, 28.50 ± 1.44) locations experience less no. of gen. in FDP. Similar incidence was followed in these locations in DP and NP.

The interaction between RCPs x location; RCPs x time slice; location x time slice and RCPs x location x time slice were significant with respect to annual gen. time and seasonal gen. time. With annual and seasonal no. of gen., the interaction was significant with RCPs × time slice; location x time slice only (Table 1). During crop growing season, Coimbatore (8.49 ± 0.28, 21.21 ± 0.06) and Mohanpur (7.81 ± 0.22, 23.73 ± 0.40) locations would experience more no. of gen. (F_10, 3828_ = 3655.79, *P* < 0.001) with reduced gen. time (F_10, 3828_ = 4498.44, < 0.001). Moderate levels were estimated for Jabalpur (7.23 ± 0.31, 24.26 ± 0.99) and Gulbarga (7.21 ± 0.31, 26.19 ± 1.08). Ananthapur (6.79 ± 0.14, 27.08 ± 0.53) and Bengaluru (6.3 ± 0.28, 30.04 ± 1.46) would have lesser no. of gen.

### Percentage variation in annual and seasonal generations

Higher percent increase of annual no. of gen. of *H. armigera* was predicted to happen in FDP period (7.65 to 37.83%) over BL period followed by DP (7.47 to 21.89%) and NP (5.25 to 9.51%) across 4 RCPs (Sup Figure 1 and 2). Similarly, the expected increase of seasonal no. of gen. over the baseline was highest in FDP period (7.07 to 35.00%) followed by DP (7.09 to 20.31%) and NP (5.00 to 8.77%) in four RCPs. Higher reduction of annual gen. time of *H. armigera* over BL period was observed in RCPs for future TSP/ CCPs. The highest reduction (percentage) in annual gen. time over BL was predicted for FDP (6.86 to 26.83%) followed by DP (6.78 to 17.67%) over NP (3.69 to 8.96%) in four RCPs. The highest percent reduction was noticed with RCP 8.5 over other three scenarios. Similar trend was with seasonal time and the percent reduction was greater in FDP (4.87 to 25.77%) followed by DP (4.70 to 18.37%) and NP (3.78 to 10.42%) over the baseline.

### Partitioned variation

**Figure 5:**
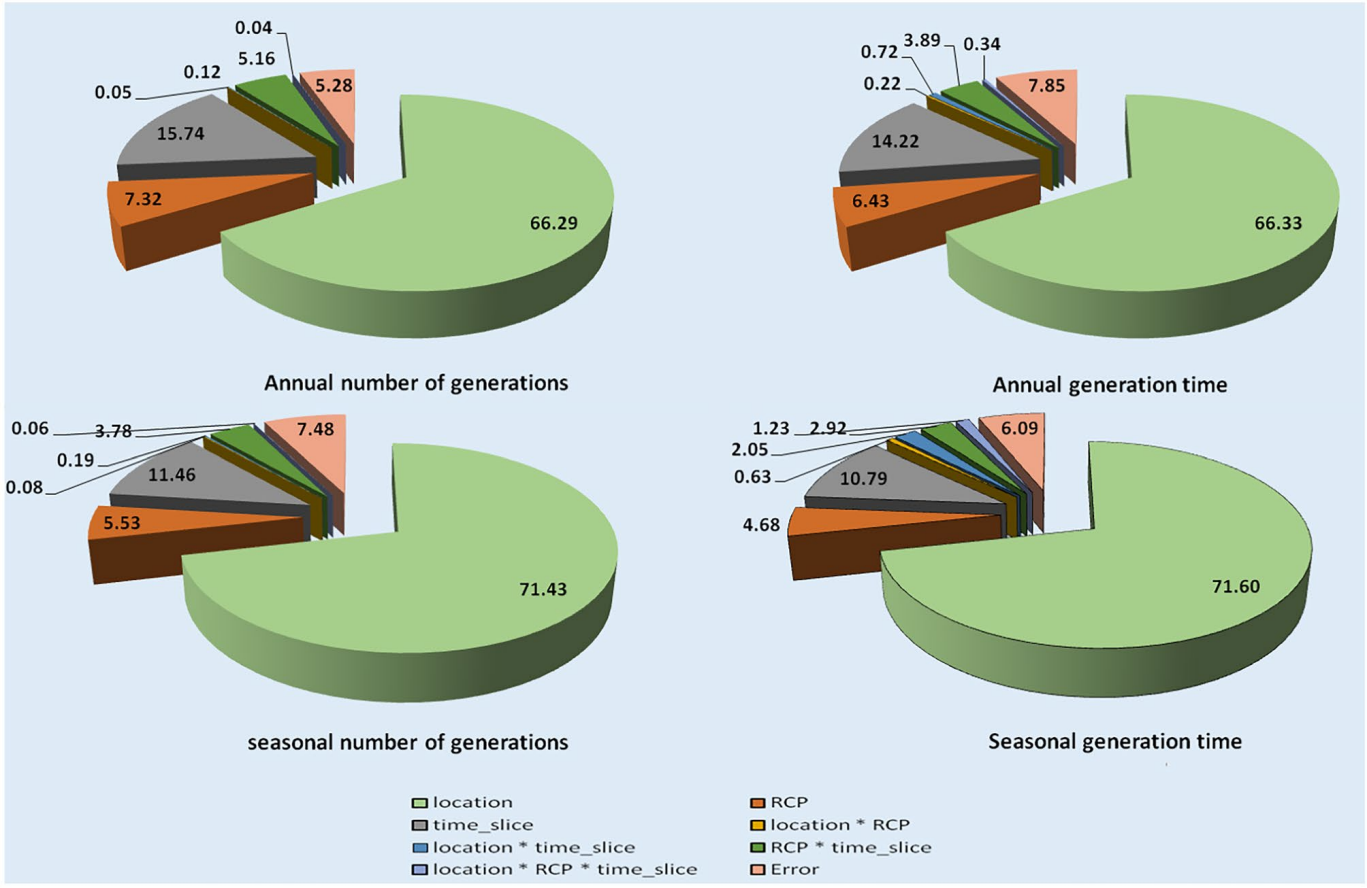
Estimated proportion of variation in predicted no. of gen. and gen. time of *H. armigera* by different variables.

Geographical location (66.29%) and time period (15.74%) were the key sources of variation in annual no. of gen. of *H. armigera*, which together explained about 82.03% of the total variation. Rest of the variables viz., scenarios and interactions contributed less than 20% of the variation (17.97%). Similarly, in annual gen. time of *H. armigera*, geographical location (66.33%) and time period (14.22%) explained most of the variation (80.55%) (Fig. 5). Similar trend was with seasonal no. of gen. and seasonal gen. time of *H. armigera* where geographical location and time period together accounted for 82.89% and 82.39% of the total variation respectively.

### Trend in generation time (seasonal) under different RCPs

**Figure 6:**
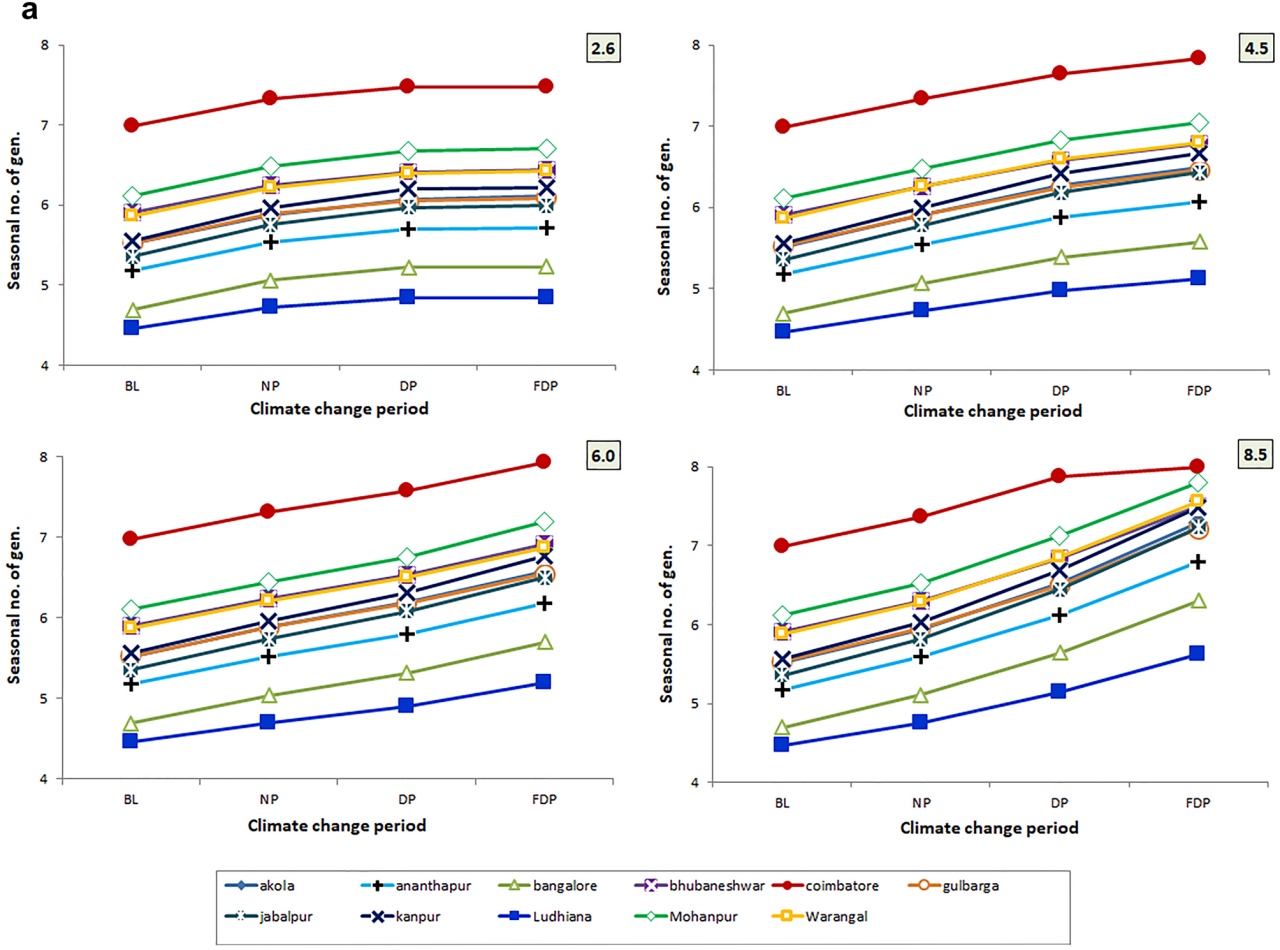

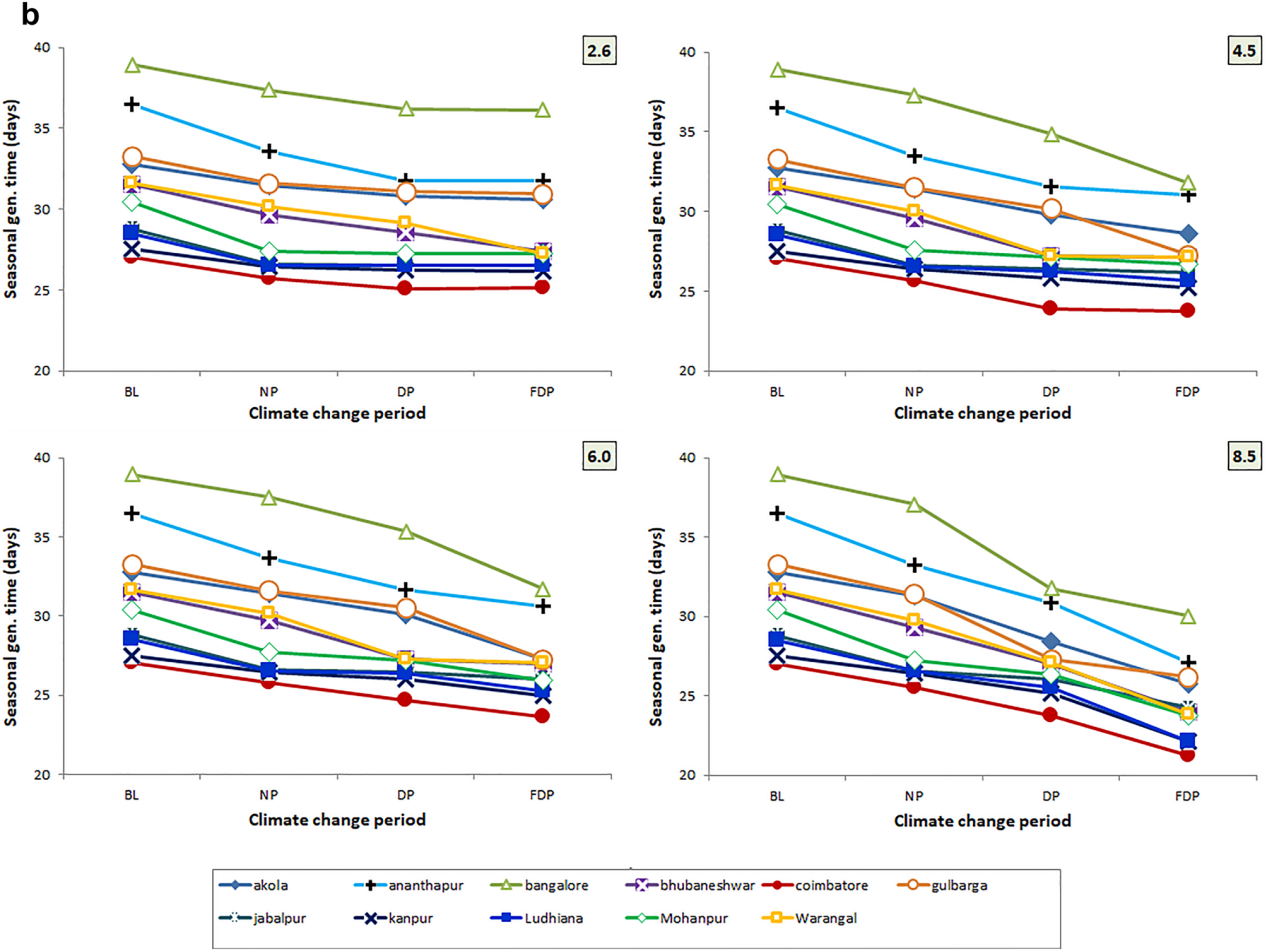
(**a**) Trend in no. of gen. in crop season across 4 RCPs and 4 CCPs. (**b**) Trend in gen. time in crop season across 4 RCPs and 4 CCPs.

Among four RCPs, the higher reduction of gen. time is noted in RCP 8.5 scenario followed by 6.0 and 4.5 scenarios. Across three CCPs, it is more evident in FDP, DP climate change period than NP period. The trend is more reflected in Coimbatore location followed by Kanpur and Ludhiana locations and least reflection was recorded in Bengaluru and Ananthapur locations across 4 RCP scenarios (Fig. 6a, b).

### Variability in seasonal generation time across locations, CCPs and RCPs

**Figure 7:**
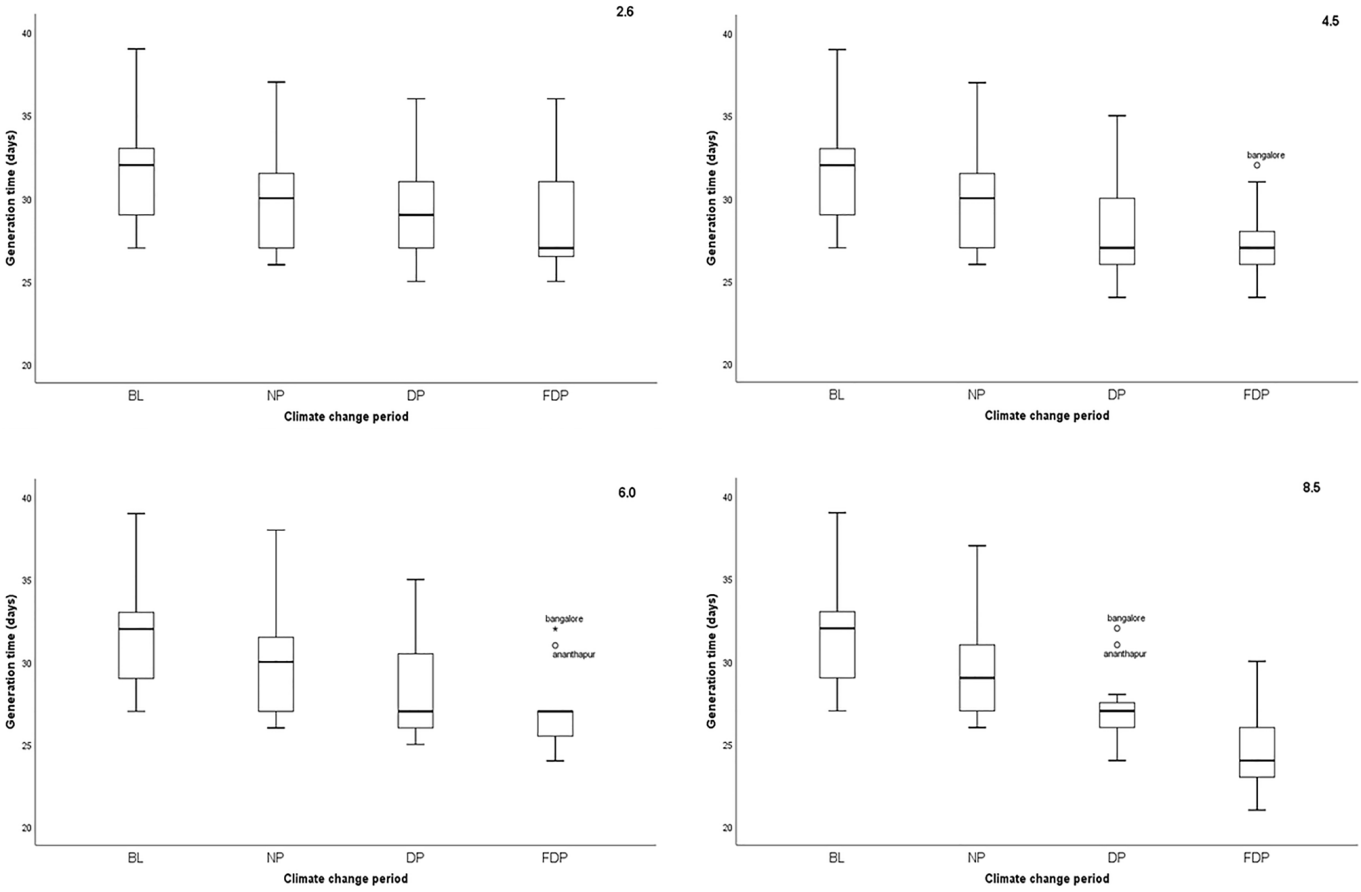
Trends in mean gen. time of *H. armigera* in Crop Season across 4 RCPs and 4 CCPs.

The changes in mean seasonal gen. time of *H. armigera* in eleven locations across different CCPs and RCPs were depicted as ‘box plot’ figure capturing the variability in the parameter and here every point indicates the mean of seasonal generation time of the 11 locations studied across 4 CCPs. The seasonal gen. time followed a decreasing trend *i.e.,* lesser gen. time with advancing climate period in 4 RCPs based on comparing the medians. The lowest gen. time recorded was 21 days in FDP of RCP 8.5 as against 39 days in BL (Fig. 7). The Outliers, Ananthapur (31 days) and Bengaluru (32 days) in FDP of RCP 4.5 and RCP 6.0 were higher than the maximum range. Similar reduction in generation time was noted in annual number of generations also.

### Generations in altered duration of pigeonpea

#### Reduction of duration

**Figure 8:**
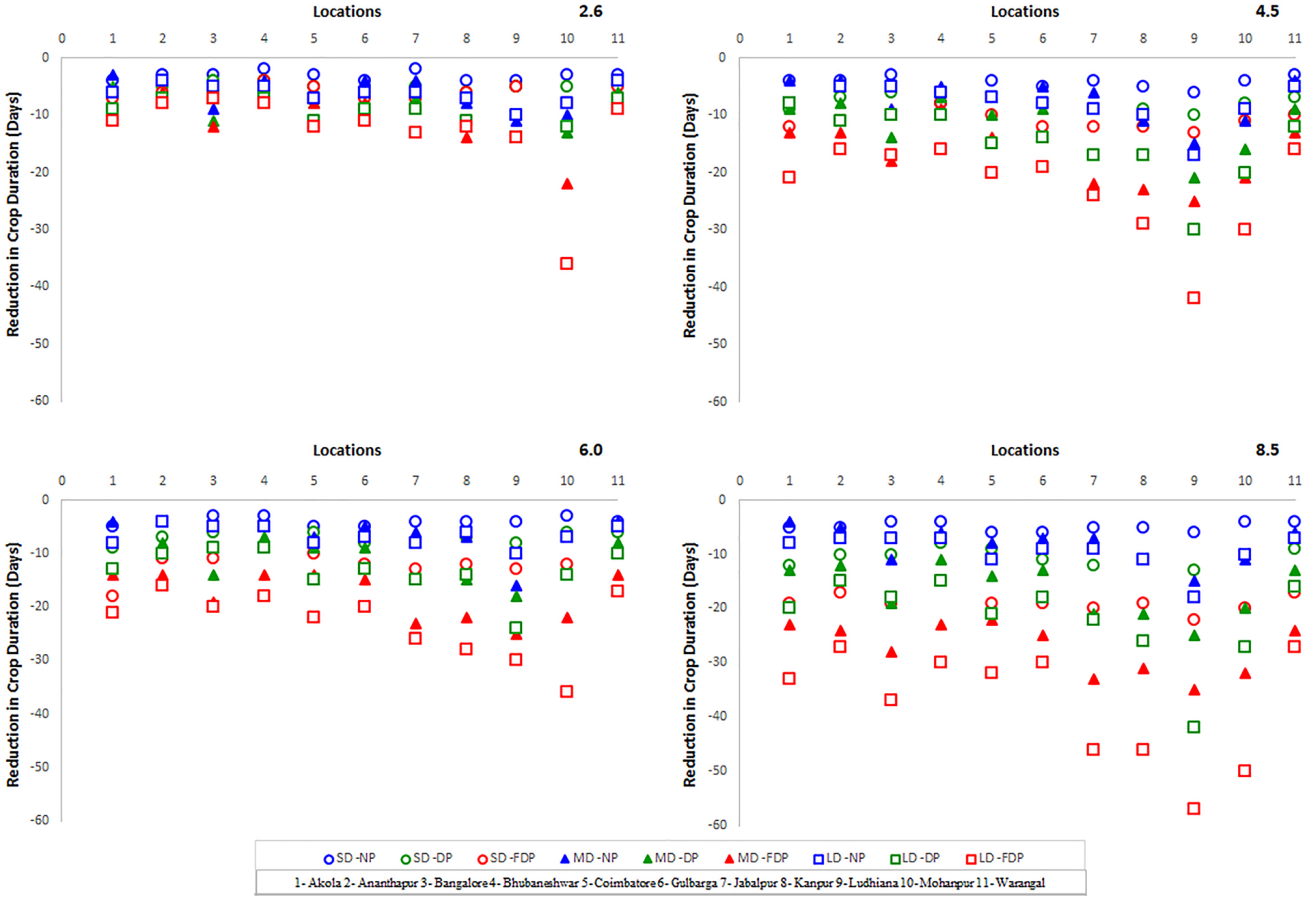
Alteration of duration of 3 pigeonpeas based on GDD across four RCP and 3 CCPs across 11 locations.

The reduction of pigeonpea duration was expected to occur across three CCPs based on GDD of 1900–2700 for SDP, 2100–3000 for MDP and 2450–3550 for LDPs at 11 locations. The data depicted in Fig. 8 indicated the reduction of crop duration across 11 locations and might be due to advancement of crop maturity.

The reduction of crop duration (days) in SDP (2–22), MDP (3–35) and LDP (5–57) was substantial at all locations across 4 RCPs and 3 CCPs and was more evident in RCP 6.0 and 8.5 (Fig. 8). The higher reduction of crop duration was in LDP (57 days) and MDP (35 days) in far distant period (FDP) of RCP 8.5 at Mohanpur and Ludhiana locations. Similar reduction of duration was reflected in other locations in DP and NP also in RCPs of 6.0 and 4.5. The reduction of crop duration was moderate at Coimbatore and Akola and least at Ananthapur, Bhubaneshwar and Warangal locations in RCP 6.0 and 8.5 scenarios (Fig. 8). Though the reduction of duration was noted in SDP, it was meagre in comparison with LDP and MDPs.

#### Number of generations in altered duration

**Figure 9:**
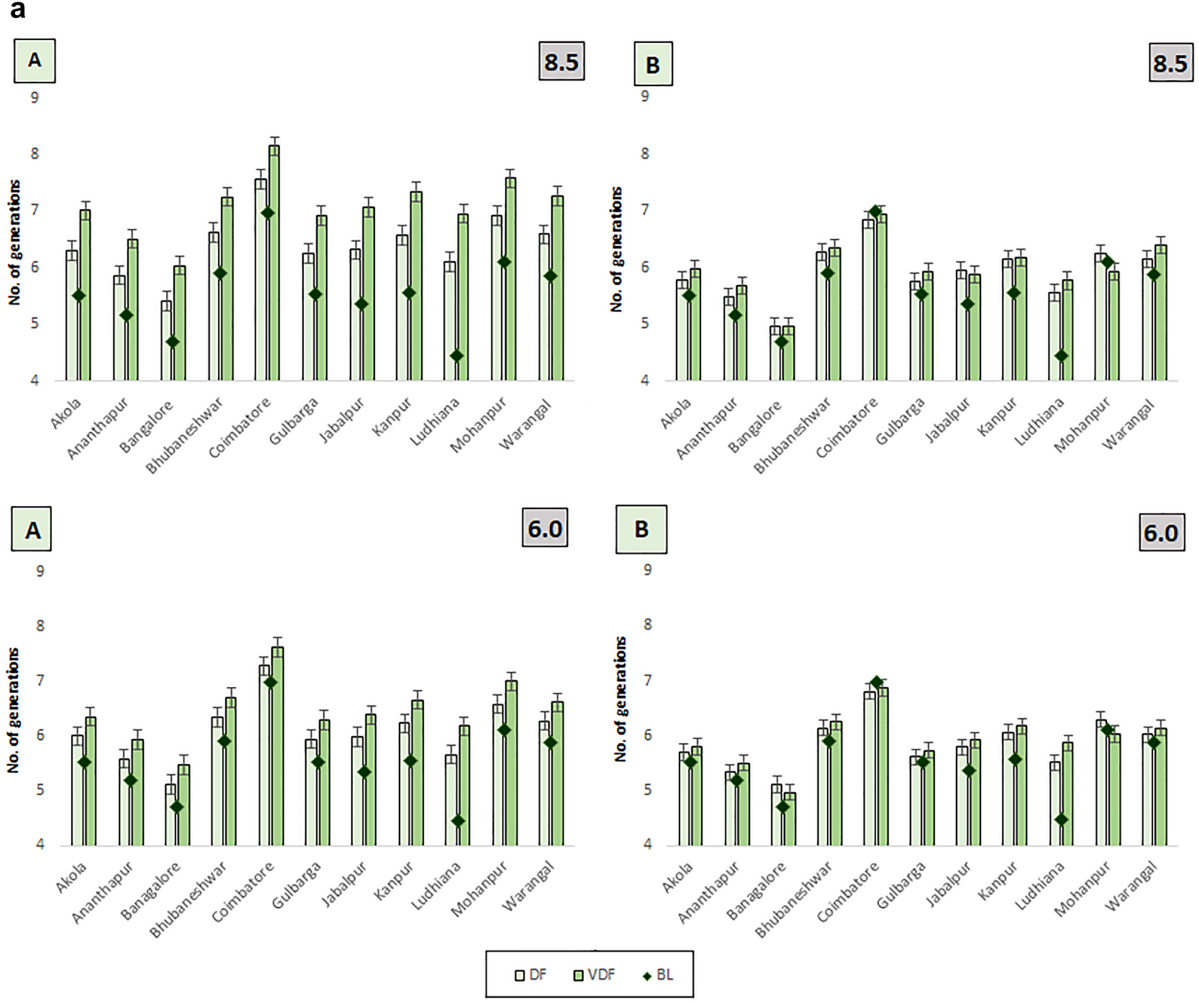

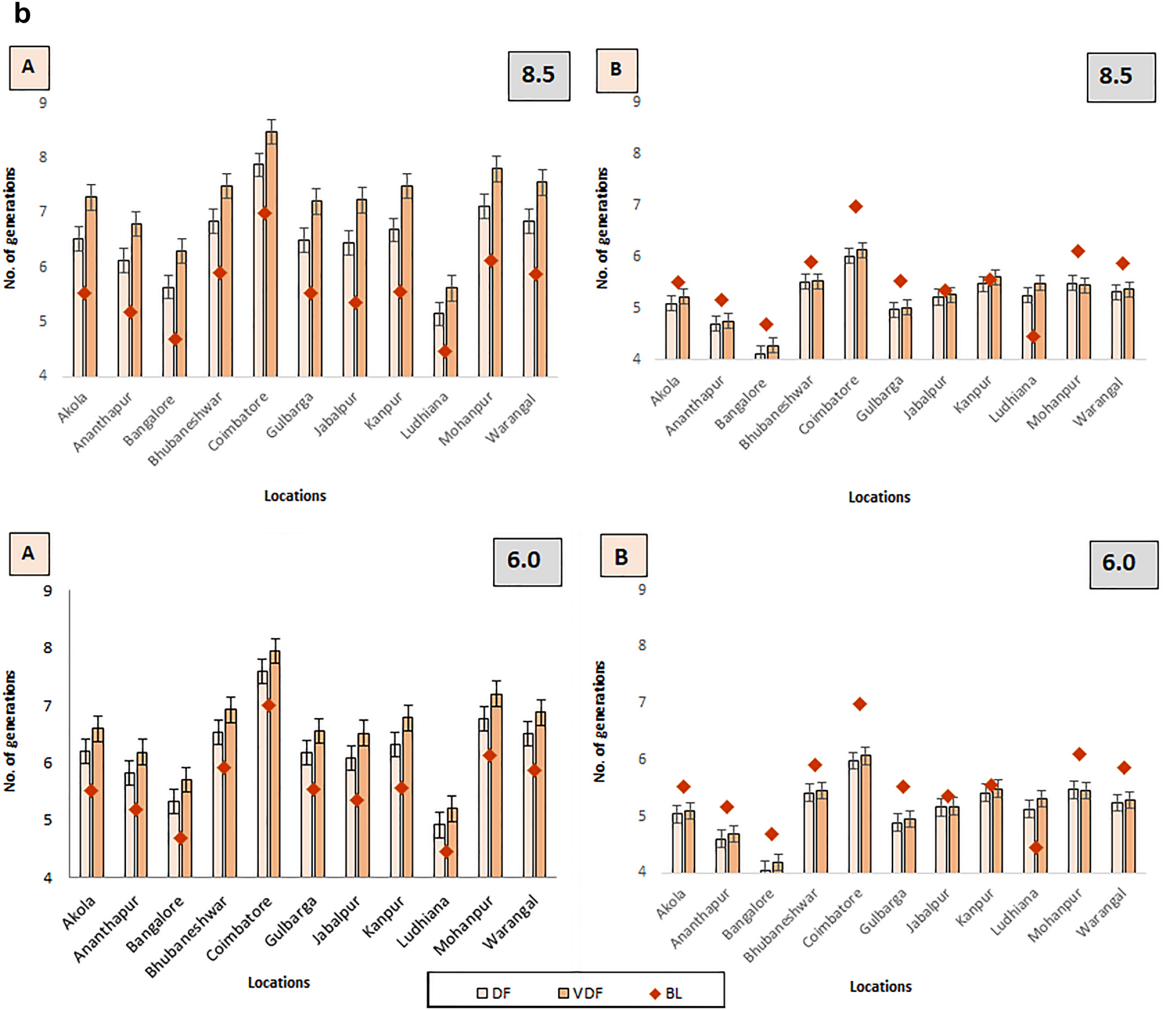
(**a**) Variation in the number of generations with altered duration in LDP during CCPs in 4 RCPs across different locations of India. (**b**) Variation in the number of generations with altered duration in MDP (**b**) during CCPs in 4 RCPs across different locations of India.

Figure 9a depicts changes in the number of generations *of H. armigera* on LD pigeonpea possible with RCP 6.0 and 8.5 scenarios. There was an observed increase in the number of insect generations even with the shortened duration of LD pigeonpea during FDP and DP for both RCP 6.0 and 8.5 with the changes being more conspicuous with RCP 8.5-based projections. At most of the locations, it was predicted that even with a reduced duration of LDP, a greater number of generations were expected to occur in both scenarios and the variation was more pronounced at Ludhiana (5.77). However, the changes in the number of generations are relatively low in the case of MD pigeonpea in most locations (Fig. 9b). In the case of SDP, not much difference in no. of gen. was recorded in RCP 2.6 and 4.5 scenarios due to reduction of crop duration meaning that the reduced crop duration may offset the accelerated rate of reproduction.

## Discussion

Increase in temperature causes advancement of insect developmental stages which in turn results in reduction of duration of life cycle and thus more generations of insects may occur in shorter period of time. The role of temperature is vital and impactful on insects in completing the life cycles and producing more generations^43^. Formulation of effective pest control measures needs complete comprehension of population dynamics of insect pests. The mean annual maximum temperature increased at the rate of 0.018 °C per year in the past 20 years and Arora et al.^44^, noted that mean temperature increased by 0.42 °C in the last 100 years. It is implied that more insect population is function of more generations would occur in a calendar year and growing season. Nationwide rise in temperature (Tmax and Tmin) was predicted across the four RCPs^45^. The increases in temperature reported by Chaturvedi et al.^3^ were about 1.7–2.0 °C by 2030s in RCP 4.5 and RCP 6.0, with overall increase in temperature for India by 1–4 °C.

Results of the present study also exhibit a similar rise in temperature (Tmax and Tmin) across eleven pigeonpea locations of India in Tmax (0.7–4.7 °C) and Tmin (0.8 to 5.1 °C) over the baseline during NP, DP and FDP of 4 RCP scenarios. We predicted the pest scenario i.e., no. of gen. of *H. armigera* using the projected temperature data from four RCP scenarios by adopting standard GDD approach in three future TSPs viz., NP, DP and FDP at eleven pigeonpea growing locations in India. There was a similar increase of temperatures for other parts of Asia^46^ by 2.06–3.63 °C and for other parts of the world^47^ by 2 °C and 6 °C.

Srinivasa Rao et al.^15^ predicted the no. of gen. of *H. armigera* on pigeonpea with temperature data of MarkSim, GCM multimodel (7) data of three scenarios (A1B, A2 and B1) of CMIP3 at eight pigeonpea locations of India. Here, we used RCP-based climate projections of CMIP5 group of climate models with several advantages^34,48^. We used temperature data of RCP scenarios as they eliminate or reduce the uncertainty (http://www.cawcr.gov.au/projects/Climatechange) in projections over the earlier ones based on SRES^49^ and RCPs have greater applicability in Indian conditions. Further, we estimated the possible reduction in duration of LDP, MDP and SDP and hypothesized that the no. of gen. would vary with reduction of crop duration among three pigeonpeas, while in our earlier study^15^, we considered a constant crop duration. In the present study, we attempted the estimation of the number of generations with altered crop duration also. Different degree day models and quantified equations/relationships are often adopted^50^ to predict the pest scenarios.

Several studies^15,16,29,30,34,37,42,51,52^ predicted the pest status in different climate change scenarios using GDD and pest modelling approach and mentioned that the pest scenario is a function of emission scenarios, geographical location, climate change period and model adopted. The present paper predicted the status of *H. armigera* on pigeonpea during future CCPs using RCP projected temperature ensemble data across India, as little or no studies have been attempted so far.

Insects are poikilothermic and thermo-sensitive, and their distribution and abundance get influenced by the smaller variations of temperature^53^. Temperature is the key factor which affects the growth and development of crop plants and insect herbivores remarkably. GDD approach was adopted earlier^16,54,55^ for prediction of pest status.

More no. of gen. per year in multivoltine species^56^ was due to the considerable increase in surface temperatures and similar findings were with lepidopteran pests *Endopiza viteana*^57^ and *Paralobesia viteana*^58^. Our present findings showed that more number of annual generations of *H. armigera* on pigeonpea (6 to 38%) would be in RCP 8.5 followed by 6.0, 4.5 over 2.6 RCPs and parallel trend is expected in growing season also. Several research workers^15,42,43,56,58,59^ also found increased number of insect generations with increase in temperature.

Shortened gen. time was noticed in all four RCPs in both the approaches adopted by us viz., calendar year (4–27%) and growing season (4–26%) and the trend was more evident in RCP 8.5 of FDP over BL. Similar reduction of gen. time with increased temperature was in *Lymantria dispar*^60^, *Brachmia macroscopa*^61^ and *Athetis dissimilis* on maize^62^. Our findings using temperature data of seven different models of Marksim indicated the occurrence of 1 to 2 additional generations with reduced gen. time of *S. litura*^29^ on peanut and *H. armigera*^15^ on pigeonpea. It was mentioned that adaptation of insects would be quicker to climate change due to short gen. time^63^ and the same is reflected in present findings. Direct influence of increased temperature on metabolic rate and developmental rate of insects^64^ and multivoltine insects are believed to take advantage of climate warming. Present findings are in tune with Altermatt^18^ who observed that increased temperature causes advancement of phenology, which in turn lead to more no. of gen. of an insect.

Among the three variables studied viz., emission scenarios, TSPs and locations, the no. of gen. and gen. time varied significantly among the locations. Among the locations, Coimbatore would experience higher seasonal no. of gen. (7.33–8.48) followed by Mohanpur (6.49–7.805) Warangal and Akola locations over the BL period of the location across the four RCPs and TSPs. The increment is least in Ludhiana (4.72–5.62) (Fig. 6a). Climate change caused the variation in spatial distribution of different insect pests among different provinces^28,65^. More crop losses are expected due to higher no. of gen. during the crop season or calendar year. No. of gen. shows the hastening of reproductive events which in turn affect the host-insect herbivore interactions implying the increased level of incidence^18^. Increased voltinism of insect pest may lead to higher infestation^66^. Often, voltinism may amplify the outbreak of pest species^67^ also. Though occurrence of no. of gen. is dependent on several factors, the role of increased temperature is vital and often leads to higher developmental rate. The preference of the host by the insect, the presence of natural enemies and other collateral factors also influence the voltinism which are not covered by the *ingen* software, however, it takes care of temperature-driven influences. Though the impact is confounding in nature, temperature plays a major and significant role and the same is captured in the present study. The findings of present study indicate higher percent increase in no. of gen. (6–38%) with reduced gen. time (7–27%) of *H. armigera* in RCP scenario 8.5 during 3 CCPs in both season and calendar year at majority of pigeonpea locations of India which are higher than earlier studies by Srinivasa Rao et al.^15^, with Marksim model data.

The results on analysis of partitioned variation indicated significant contribution (about 83%) (Fig. 5) of geographical location and time period together in the variation in annual and seasonal no. of gen. and annual and seasonal gen. time indicating their vital role in prediction of the same. Pest modeling is most practical and useful method for predicting the weather impacts on insect pests^68^. Our findings indicated that temperature variation is evident across geographical locations of India among various climate change scenarios meaning that temperature impacts on insects vary with location. Pest modelling is the most adopted tool to quantify these impacts which are location and species specific. Present findings indicated that 83 percent of the total variation in projected no. of gen. of *H. armigera* on pigeonpea is explained by geographical location and climate period only. Similar findings were stated by Ziter et al.^42^ and Srinivasa Rao et al.^15,29^. The contribution of these two variables to total variation in our present studies was lesser than our earlier studies^29^ which accounted for about 94%. This might be due to the adoption of RCP scenario data which has more reliability for India and had about 7–11% of the total variation. The interactions among three variables also contributed substantially though they are in less percentages.

It is documented that at higher temperatures and warming conditions, advancement of crop phenological stages would take place resulting in reduction of crop duration^69–71^. Earlier authors^38–40^ justified the advancement of phenological stages of crops and maturity with increased temperature causing variation in thermal requirement of pigeonpea. The present findings also indicated the reduction of crop duration based on Growing Degree Days and noted that the advancement of crop maturity was considerable in MDP and LDP and the reduction of crop duration was higher in medium (3–35 days) and long (5–57 days) duration pigeonpeas as against SDP (2–22) at 11 locations across 4 RCPs and 3 CCPs (Fig. 9). Similar reduction of pigeonpea duration by 10 days with 2 °C rise in temperature was found in Gulbarga region^72^. The results are in conformity with those of Purnamawati et al.^41^ who also reported the reduction of crop duration under increased temperature conditions. The present findings indicate clearly that reduction of crop duration across three pigeonpeas was considerable at increased temperature conditions in 4 RCP scenarios of 3 CCPs.

Even with reduced crop duration, higher no. of gen. with shortened gen. time was expected in LDP and MDP in far distant period (5.48 to 5.86; 22.17 to 22.76) than distant and near periods and was more evident in RCP 8.5 scenario. In case of SDP, no much difference in no. of gen. and gen. time was noted in RCP 2.6 and 4.5 scenarios and in NP period. An increase in the number of generations of *H. armigera* was predicted to occur even with the reduced duration of LD pigeonpea during FDP and DP for both 6.0 and 8.5 RCP scenarios and was more apparent with later scenario. Similar trend was with most of the locations and more evident at Ludhiana location. Whereas in MD pigeonpea, the change in the number of generations was found to be relatively low. Present findings indicated that the reduction of crop duration may offset the accelerated rate of reproduction of *H. armigera* in MD and SD pigeonpeas. Thus, the adoption of LDP has to factor in the higher need for pest management. The phenology of various ecological processes was potentially sensitive to climate change as influenced by temperature^20^ and advances the phenology of the *H.* armigera^73^ and similar trend was noted in the present findings.

## Conclusions

Increase in mean atmospheric temperature can often influence insect generations by causing the reduction in gen. time. The present findings predict more no. of gen. of *H. armigera* at eleven pigeonpea growing locations during future TSPs across four RCPs. Similar trend is expected with altered crop duration of pigeonpea also. Pest scenario is not only driven by temperature but also other factors like elevated CO_2_, differential rainfall, crop phenology and different trophic interactions. The present findings captured the significance of increased temperature and its impact on pest scenario only and the availability of complete dataset on other parameters may give the clear-cut comprehension of impact of climate change.

## Supplementary Information

Supplementary Information.

## Data Availability

The datasets used and/or analysed during the current study available from the corresponding author on reasonable request.
