# Supplementary material for: Pest scenario of Helicoverpa armigera (Hub.) on pigeonpea during future climate change periods under RCP based projections in India
Source: Sci Rep. 2023 Apr 26;13:6788. doi: 10.1038/s41598-023-32188-1 (PMC10133267; doi:10.1038/s41598-023-32188-1)

Supplementary Table S1. Number of locations (out of 11) to which data are homogeneous as compared to BL across 4 RCPs and 3 CCPs under study after Levene Test

Parameter	RCP 2.6			RCP 4.5			RCP 6.0			RCP 8.5		
	NF	DF	VDF	NF	DF	VDF	NF	DF	VDF	NF	DF	VDF
**Annual GT**	11	11	10	10	9	10	10	11	11	11	11	11
**Annual NG**	11	11	11	11	11	11	11	11	11	11	11	11
**Seasonal GT**	9	10	11	10	10	11	11	11	11	11	11	11
**Seasonal NG**	11	11	10	11	11	10	11	11	11	11	11	11
Total	42	43	42	42	41	42	43	44	44	44	44	44
11 locations x 4 RCPs x 3CCPs= 528												
**Total**	**515/528**											

Sup Fig 1. Per cent change in no. of gen. of *H. armigera* during 3 CCPs over baseline across 11 pigeonpea growing locations of India (Supplementary figure 1)

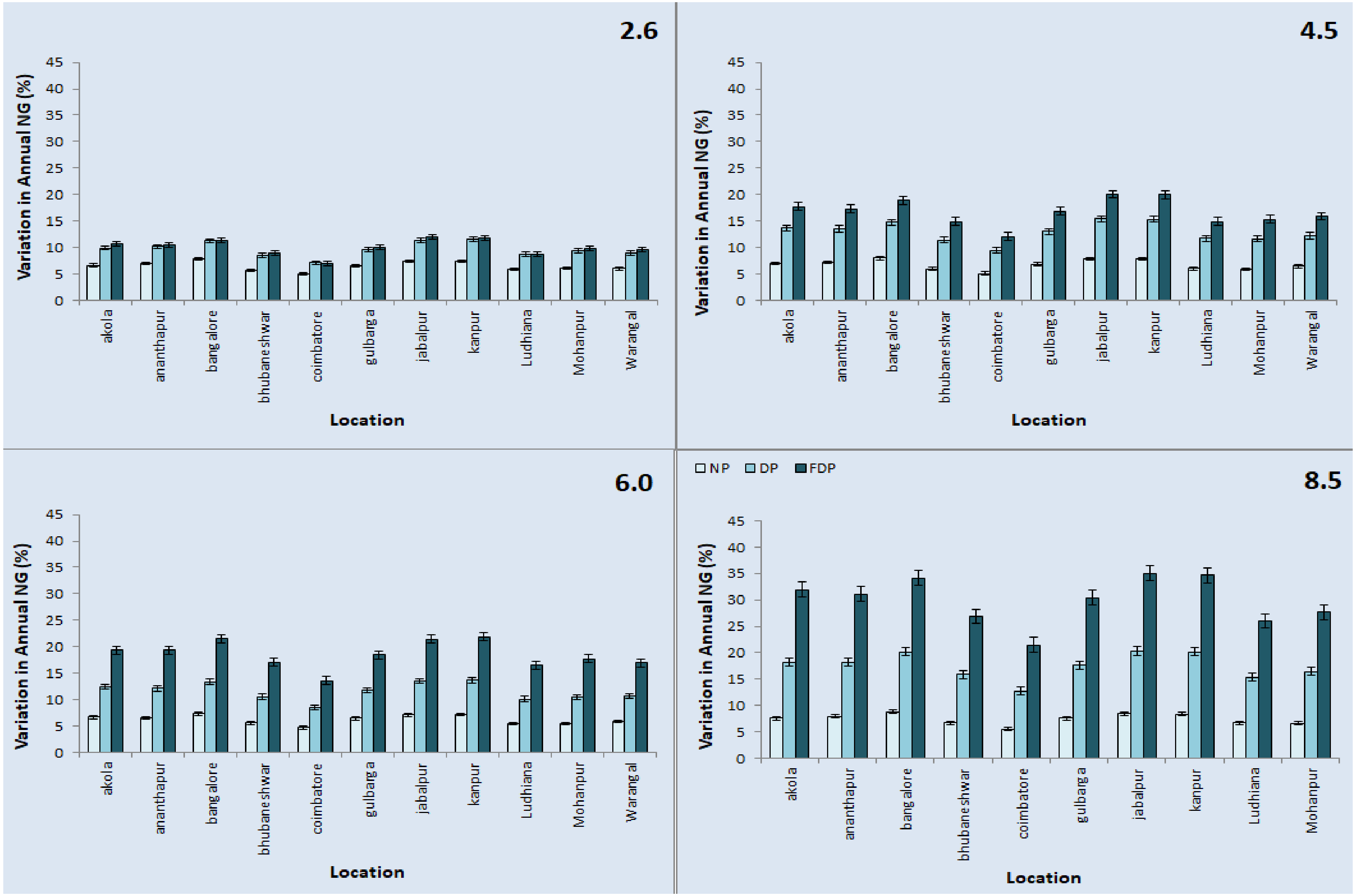

Sup Fig 2. Per cent change in gen. time of *H. armigera* during 3 CCPs over baseline across 11 pigeonpea growing locations of India (Supplementary figure 2)

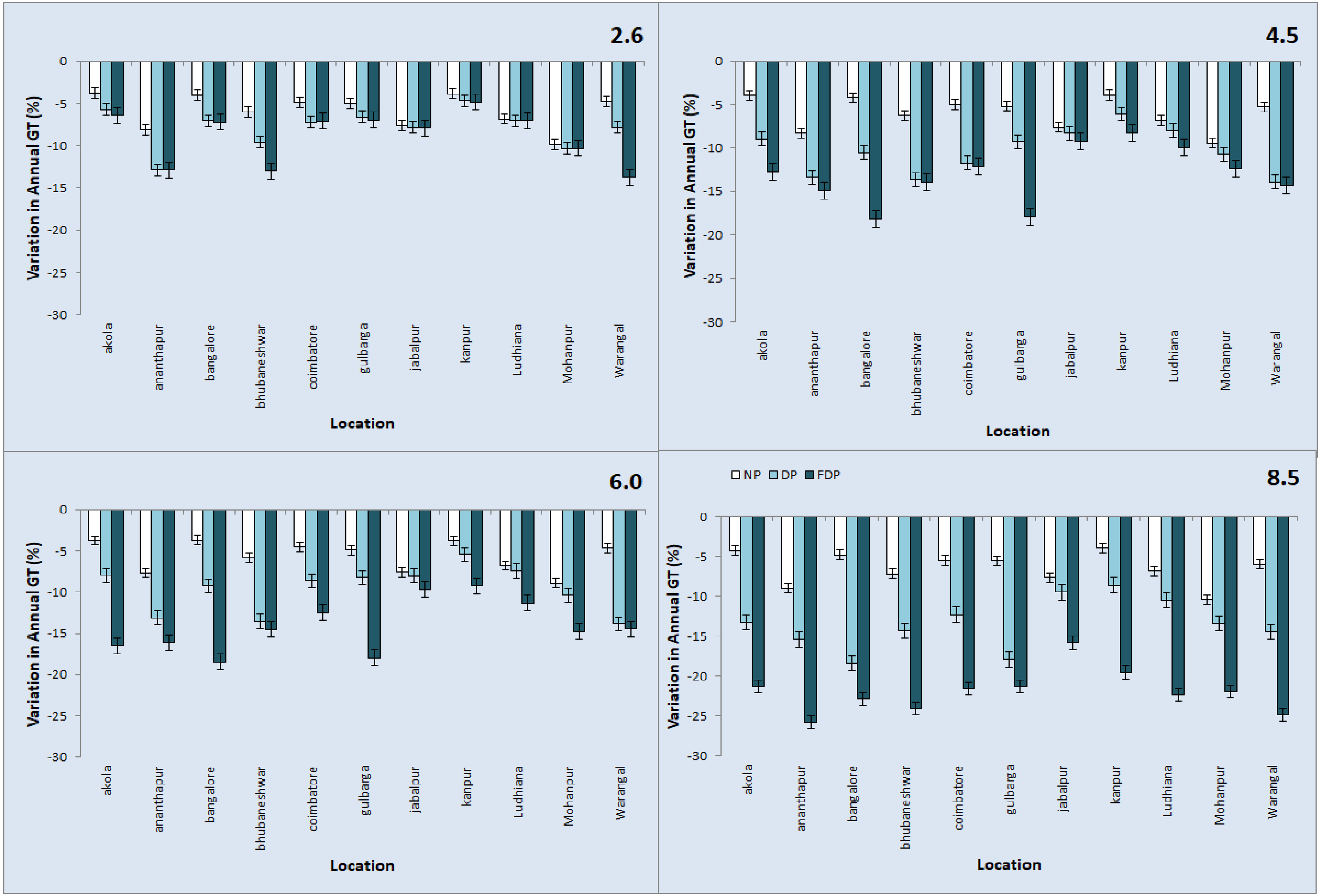

Supplement: Supplementary file 1 — Supplementary Information. [file 41598_2023_32188_MOESM1_ESM.pdf]
